# Preparation and Performance of Biodegradable Poly(butylene adipate-*co*-terephthalate) Composites Reinforced with Novel AgSnO_2_ Microparticles for Application in Food Packaging

**DOI:** 10.3390/polym15030554

**Published:** 2023-01-21

**Authors:** Raja Venkatesan, Krishnapandi Alagumalai, Seong-Cheol Kim

**Affiliations:** School of Chemical Engineering, Yeungnam University, Gyeongsan 38541, Republic of Korea

**Keywords:** poly(butylene adipate-co-terephthalate) (PBAT), AgSnO_2_ microparticles, composites, biodegradable, antimicrobial activity, food packaging

## Abstract

Biodegradable composites with antimicrobial properties were prepared with microparticles of silver stannate (AgSnO_2_) and poly(butylene adipate-co-terephthalate) (PBAT) and tested for applications in food packaging. The PBAT matrix was synthesized and confirmed by ^1^H-nuclear magnetic resonance spectroscopy, Fourier transform infrared spectroscopy, and X-ray diffraction (XRD). Ultrasonic and coprecipitation methods were used to synthesize AgSnO_2_. A two-step mixing method and a solvent cast technique were utilized to fabricate the PBAT composites (different weight % of AgSnO_2_) for packaging foods. Attenuated total reflection-infrared spectroscopy, X-ray photoelectron spectroscopy, XRD, and scanning electron microscopy were used to investigate the formation, structure, and size of the composites. Thermogravimetric analysis and differential thermal calorimetry were used to examine the PBAT/AgSnO_2_ composites. The best characteristics are exhibited in 5.0 wt. % AgSnO_2_ loaded PBAT composite. The tensile strength, elongation at break, water vapor transmission rate, and oxygen transmission rate were 22.82 MPa, 237.00%, 125.20 g/m^2^/day, and 1104.62 cc/m^2^/day.atm, respectively. Incorporating AgSnO_2_ enhanced the hydrophobicity of the PBAT materials as evaluated by the water contact angle. The 5.0 wt. % AgSnO_2_/PBAT film shows a favorable zone of inhibition against the bacteria pathogens *S. aureus* and *E. coli*, according to an evaluation of its antimicrobial activity. The weight loss of 5% AgSnO_2_/PBAT film was 78.4% after eight weeks in the natural soil environments. In addition, the results of food quality studies recommend that AgSnO_2_/PBAT (5.0 wt. %) film had a longer food shelf life than the neat PBAT and commercial, increasing it from one to 14 days for carrot vegetables.

## 1. Introduction

The development and increasing utilization of plastic products in a broad range of industries has contributed to their growth as one of the most significant materials of the 21st century. The majority of these plastics, such as polyethylene (PE), polypropylene (PP), and polystyrene (PS), among others, were produced from petroleum, and as a result, their wastes cannot degrade. Moreover, the increasing use and fabrication of plastic materials has the unintended consequence of growing oil usage and significantly affecting the environment. The two main issues caused by traditional plastics, the use of fossil energy and environmental pollution, should be resolved by forthcoming sustainable development [1]. Biodegradable plastics can be decomposed by bacteria, fungi, and other naturally occurring microorganisms. Accordingly, biodegradable plastics are in demand because the waste produced can be recycled by microorganisms in a natural environment. Due to their rapid growth and widespread use in recent years, biodegradable polymers are recognized worldwide as a solution to these plastic issues. Fresh fruit and vegetable packing is one of the most critical facets of the supply chain that extends from the manufacturer to the end user. Fresh produce packaged in antimicrobial films for protection can have an improved shelf life [2,3]. Furthermore, the pathogens that contaminate the food and bacteria that spoil food can be controlled [4,5]. These packages are often increased by combining antimicrobial agents that are adsorbed or applied to the packing surface of a material as a coating. While antimicrobial packaging films and other compounds have been investigated for a long time, their effectiveness is limited by numerous manufacturing restrictions. The growth of the market for biodegradable materials for packaging can be attributed to their environmentally friendly use and sustainable development [6,7]

Poly(butylene adipate-co-terephthalate) (PBAT) is a flexible synthesized aliphatic-aromatic copolyester with outstanding chemical and hydrophobic characteristics. The material is biodegradable and, with the help of natural sources of enzymes found in soil, can be fully decomposed within a few weeks [8,9,10]. These characteristics make it a special eco-friendly polymer that can be used in the food packaging industry of non-biodegradable polymers in garbage bags, carrier bags, cling, and mulch films [11,12]. Although PBAT is biodegradable, its poor performance limits its applications. Furthermore, compared to commonly used polymers, the cost of the PBAT is somewhat high. PBAT has been investigated widely among biopolymers because of its admirable physical and mechanical characteristics [13,14]. PBAT, an attractive polymer matrix for packaging, has improved due to its good mechanical and optical characteristics [15,16]. On the other hand, PBAT has significant drawbacks restricting its use in food packaging, such as a slow crystallization rate [17], and fewer barrier properties [18]. The use of nanoparticles (NPs) in food packaging has increased to overcome these issues and support PBAT. Previously, the PBAT material was incorporated with some nanoparticles, including TiO_2_ [19], ZnO [20], Ag_2_O [21], SiO_2_ [22], SnO_2_ [23], MgO [24], and MgO/Ag [25]. AgSnO_2_ is the most preferred metal oxide nanofillers because of its unique characteristics, such as non-toxicity, large surface area, high mechanical strength, and crystallization-inducing abilities [26,27,28]. On the other hand, there are two issues with the development of AgSnO_2_ materials. The first is that SnO_2_, a transparent bandgap semiconductor material, has poor wettability with liquid Ag, making it simple to form an insulating material, resulting in large contact resistance and temperature.

Recent studies on biodegradable composites prepared by solution casting and containing nanofillers are limited [29,30]. A solution cast technique was used to fabricate composites of PBAT and different wt. % of AgSnO_2_. Scanning electron microscopy (SEM) showed that AgSnO_2_ has a planar single-layer structure, and Ag and Sn interact extensively. AgSnO_2_ are dispersed uniformly within the PBAT because of the good compatibility and interaction with the AgSnO_2_ and PBAT, in accordance with the microstructure of the film surface. The tensile strength of the PBAT composite increased to 22.82 MPa with 5.0 wt.% (ACP-5.0), compared to 9.43 MPa for PBAT (ACP-0.0) without reinforcement. Thermogravimetric-differential thermal analysis (TG-DTA) indicated that the weight loss temperature (%) is significantly higher in the PBAT/AgSnO_2_ composites than in PBAT alone. In addition, the PBAT/AgSnO_2_ composite films revealed strong hydrophobicity. Since AgSnO_2_ shows synergistic antimicrobial activity, the AgSnO_2_-filled PBAT composite exhibited superb antimicrobial activity toward *S. aureus* and *E. coli*. In conclusion, those composites could be excellent for antimicrobial packaging because of their specific properties (mechanical, thermal, barrier, and antimicrobial). Therefore, PBAT/AgSnO_2_ composite films were developed and assessed for food packaging.

## 2. Materials and Methods

### 2.1. Materials

In this case, 1,4-Butanediol (BDO, 99%, TCI Chemicals, Seoul, Korea), along with dicarboxylic acids, such as terephthalic acid (PTA, 98%, Sigma-Aldrich, Saint Louis, USA) and adipic acid (AA, 99%, Sigma-Aldrich), were used as the monomers in a copolymer for the synthesis of PBAT. The melting temperature, density, and molecular weight (Mw) of the synthesized PBAT were 125 °C 1.25 g/cm^−3^ 10.2 × 10^4^ g/mol^−1^, respectively. Triethyl phosphate (TEP, 99%, Samchun Chemicals, Seoul, Republic of Korea) was used as a heat stabilizer during the polymerization reaction. Tetrabutyl titanate (TBT, 97%, Sigma-Aldrich) was employed as a catalyst during the esterification reaction. Sodium stannate (Na_2_SnO_3_) and silver nitrate (AgNO_3_) were purchased from Sigma-Aldrich in Korea. Ethylene glycol, acetone, chloroform (99%), and liquid ethanol were supplied from Daejung Chemicals (Korea). All chemicals recovered were used as received.

### 2.2. Synthesis of AgSnO_2_ Microparticles (MPs)

AgSnO_2_ was synthesized using the methodology reported elsewhere [30]. AgSnO_2_ was synthesized from a reactive metal precursor with an overall molar ratio of 1:2 using a coprecipitation procedure with ultrasonic assistance. Briefly, 0.1 M sodium stannate (Na_2_SnO_3_) and 0.01 M silver nitrate (AgNO_3_) in DI water were combined in equal volumes to a total of 60 mL. The silver nitrate was continuously stirred while the second solution of sodium stannate was added. Subsequently, 10 mL of soluble urea (3 g/mol) and an ethylene glycol capping agent were added slowly to the mixed solution with stirring. The solution was then irradiated with ultrasonic radiation in an ultrasonic bath for one hour (Ultrasonic Bath Elmasonic S10; 100 W, 37 kHz). The precipitate was filtered and washed with DI water to eliminate the reaction residues. The residual moisture and hydrate contents of the precipitate were removed from the catalytic reaction with ethyl alcohol. The obtained catalyst material followed a five-hour calcination process at 600 °C in air. The annealed AgSnO_2_ was kept in an airtight container for physicochemical analyses and used. Data from characterization were consistent with those from the previous article [31].

### 2.3. Synthesis of Poly(butylene adipate-co-terephthalate) (PBAT)

**Figure 1:**
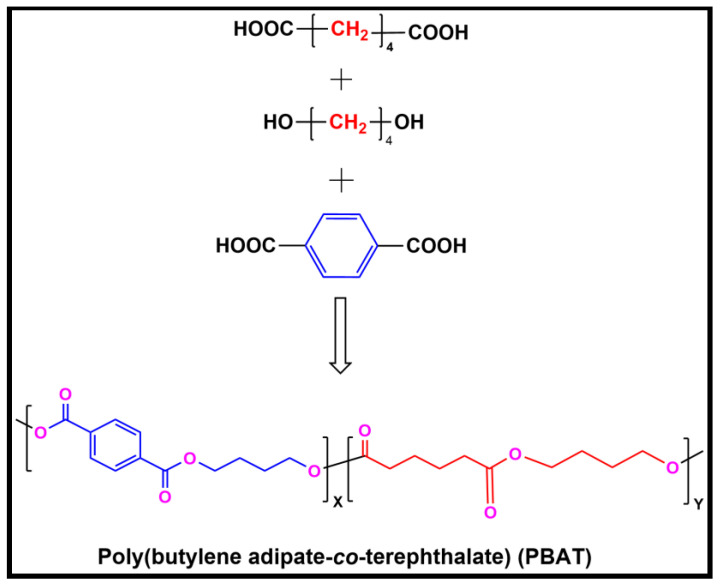
Synthesis of poly(butylene adipate-co-terephthalate) (PBAT).

PBAT was synthesized using the methodology reported elsewhere [32]. The polycondensation method is used to make polyesters from mixtures of diols and dicarboxylic acids [33]. As shown in Figure 1, using the polyester synthesis method, butanediol (BDO), terephthalic acid (PTA), and adipic acid (AA) can be used to synthesize PBAT through a copolymerization reaction. Initially, monomers, such as BDO and dicarboxylic acids (AA and TA), were used for esterification. In a reaction mixture, the main materials were added in the following amounts: AA (7.3 g, 0.05 mol), BDO (54.0 g, 0.60 mol), PTA (33.6 g, 0.20 mol), TEP (21.8 mg, 0.12 mmol), and TPT (21.3 mg, 0.06 mmol). The reaction was conducted in an Ar atmosphere at 220 °C for three hours of polycondensation. The pressure was increased to 30 torr for melt polycondensation, and the solution was heated to 240 °C at 5 °C/min. In an argon atmosphere, the material was collected and warmed to room temperature for three hours. The weight average molecular weight (Mw) of the prepolymer was 2.8 × 10^4^. The solid prepolymer was crushed and sieved through a 40-meshes to yield 2.0 g of granules. Granules of the sieved prepolymer were then placed into a solid-state polycondensation reactor and reacted for 13 h under 30 torr. The polydispersity index (PDI) and calculated Mw of the product were 1.90 and 2.40 × 10^5^, respectively. The synthesis of an aromatic-aliphatic matrix based on PBAT was accomplished by melt polymerization.

### 2.4. Preparation of PBAT/AgSnO_2_ Composite Films

Prior to use, the PBAT pellets were dried in an oven for 24 h at 60 °C. The PBAT composites are prepared using the solution mixing and drop-casting method [34,35]. After dissolving 2.0 g of PBAT in chloroform, the estimated amount of AgSnO_2_ was added. After 12 h, the stirred solutions were cast onto a glass plate and sonicated for 30 min to produce PBAT/AgSnO_2_ (ACP) composites. The antimicrobial properties of the solvent were evaluated by allowing an additional 24 h to dry at room temperature [35]. ACP-0.0, ACP-0.5, ACP-1.0, ACP-2.0, ACP-3.0, and ACP-5.0, respectively, are abbreviations for PBAT and PBAT/AgSnO_2_ composite film of 0.5, 1.0, 2.0, 3.0, and 5.0 weight %. Table S1 lists the sample specifications.

### 2.5. Characterization

#### 2.5.1. Structural Characterization

Deuterated chloroform (CDCl_3_) was used as the solvent, and tetramethyl silane acted as the reference to record the ^1^H-nuclear magnetic resonance (^1^H-NMR, Bruker instrument, OXFORD, AS600, Concord, MA, USA) (600 MHz) spectra. The Fourier-transform infrared spectra (Perkin-Elmer Spectrum Two) were recorded in attenuated total reflection (ATR)-FTIR mode in the 4000–400 cm^−1^ spectral region. X-ray diffraction (XRD, Rigaku, PANALYTICAL) was conducted at a scan rate of 0.50° 2θ min^−1^ over a scan range of 10 to 80° 2θ.

#### 2.5.2. Morphological Studies

Scanning electron microscopy (SEM) analysis was performed to observe the microstructure of the film samples. Small piece of film sample was mounted on a SEM specimen holder and analyzed using a SEM (JEOL 6400, Tokyo, Japan) with an accelerating voltage of 5.0 kV.

#### 2.5.3. Thermal Characterization

TGA (TA Instruments, SDT Q6000) was performed to determine the thermal stability. TG-DTA was performed in a N_2_ atmosphere at a scanning rate of 20 °C/min (nitrogen flow rate was 60 mL min^−1^). A 5.0 mg sample of the PBAT/AgSnO_2_ composite samples was employed in the differential scanning calorimetry tests (TA Instruments, DSC Q200). The samples were heated in a N_2_ atmosphere at a flow rate of 50 mL per minute from room temperature to 300 °C. In the case of PBAT, the thermal history was removed by the sample being heated to 180 °C and maintained for two minutes. The material was then heated to 180 °C and cooled to 50 °C. The heating rate used for all DSC runs was 20 °C/min.

#### 2.5.4. Mechanical Strength Measurements

According to ASTM-D882, the tensile strength of PBAT and its composites was tested at 23 °C and 50% RH using a universal testing machine (3345, Instron, Norwood, MA, USA). The PBAT composite used for the study was 50 mm × 20 mm in size, with a gauge length of 30 mm, and running at a 20 mm/min speed. The average was calculated by analyzing five samples for the tensile strength. The maximum tensile strength was indicated in MPa. The film thickness was determined to be within 0.001 mm using a digital thickness measurement (Mitutoyo micrometer, Tokyo, Japan). The values were determined as the mean of at least five places. The physical and mechanical characteristics of the materials were evaluated.

#### 2.5.5. Barrier Properties

According to the ASTM D3985 standard method, the oxygen transmission rate (OTR, Noselab, ATS, Italy) for the PBAT and its composites were evaluated at 23 °C and one atmosphere. The composite samples were measured at five multiple locations, and the average value was used. The specimen was handled at room temperature. According to ASTM F1249-90, the WVTR values of PBAT and PBAT/AgSnO_2_ composites were evaluated using a Lyssy L80-5000 under 23 °C. The test was repeated five times, with the average used for further analysis.

#### 2.5.6. Water Contact Angle Measurements

Using the water contact angle of the PBAT and its composites was examined using the sessile drop method with a contact angle meter (Dataphysics Instruments, OCA-20, Filderstadt, Germany) at 23 °C. To measure the contact angle, a 1 L droplet of water was placed on the surface of the composite of the films and PBAT, and an image of the droplet was recorded in 5 s. After conducting surface tension measurements at five different locations on the film, the mean value was calculated. The standard error for the test observation was 1°.

#### 2.5.7. Antimicrobial Activities

The antimicrobial activities of PBAT composite materials were tested to use the zone-of-inhibition method. With the help of a pattern, calipers, or a ruler, the zone of inhibition can be measured. Its measurements are given in millimeters, with the closest millimeter often being used. The composites conducted an advancing exposure test in accordance with the ASTM E2149-01 against Gram-negative (*E. coli*) and Gram-positive (*S. aureus*) microorganisms. To make a broth, 100 mL of water was mixed with 1.0 g of beef extract and peptone (2.0 g). For 24 h, the solution was maintained in a shaking incubator at 40 °C and 200 rpm. The microorganism cell suspension of *E. coli* and *S. aureus* was diluted by 10^6^ in a 0.90% sterile NaCl aqueous solution.

#### 2.5.8. Soil Burial Degradation Test

At a depth of 9 cm, the PBAT/AgSnO_2_ composite samples measuring 2.5 cm × 5.0 cm were buried in natural soil. The soil pH was 5.0, the temperature was approximately 30 °C, and the environmental RH was approximately 60%.

#### 2.5.9. Overall Migration Studies

The migration of AgSnO_2_ was determined by inductively coupled plasma-mass spectrometry (ICP-MS, ION-300X, PerkinElmer, Waltham, MA, USA). The EU laws (10/2011/EU) were fulfilled while performing the migration tests.

#### 2.5.10. Statistical Analysis

*ANOVA* in SPSS 21 was used (IBM, New York, NY, USA) to determine the statistical significance of each result. The data are given as the mean ± standard deviation. Statistical differences were analyzed using a one-way analysis of variance, and a value of *p* < 0.05 was considered significant.

## 3. Results and Discussion

### 3.1. Characterization of AgSnO_2_ Microparticles

**Figure 2:**
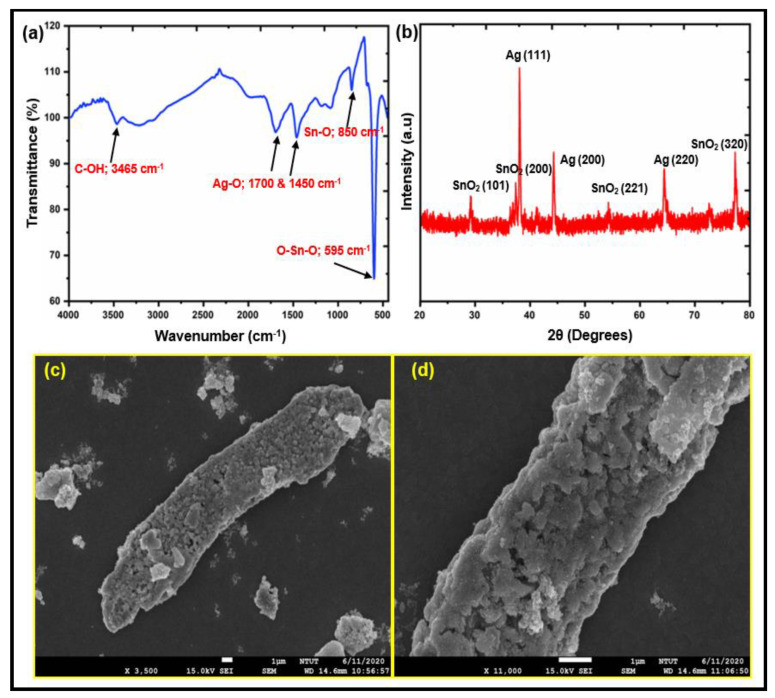
Structural and morphological characterizations of AgSnO_2_: (**a**) FTIR; (**b**) XRD; (**c**) SEM image with Lower magnification, and Higher magnification (**d**).

FTIR, XRD, and SEM images were used to describe the structure and morphology of the synthesized AgSnO_2_ microparticles, as shown in Figure 2. The characteristic absorption peaks at 950 and 570 cm^−1^ in Figure 2a were a result of the bending and stretching modes of SnO_2_ termed Sn–O and O–Sn–O, respectively. The as-synthesized AgSnO_2_ exhibited a strong absorption for the O–H stretching band at 3000 cm^−1^ in addition to weak peaks for C–OH at 1690, 1380, and 1255 cm^−1^, showing that the AgSnO_2_ contained hydroxyl groups. The Ag–O bond was indicated by the peak at 1065 cm^−1^; Ag with SnO_2_ was used to confirm this bond. Figure 2b shows the XRD patterns of the hydrothermally synthesized AgSnO_2_. The four main peaks of AgSnO_2_ were observed at 29.11°, 37.42°, 54.35°, and 77.30° 2θ, respectively. Those contributed to the (101), (200), (221), and (320) Bragg reflections of the tetragonal structure of SnO_2_, respectively. The face-centered cubic crystal structure of silver metal also revealed three strong reflections at 38.11°, 44.21°, and 64.33°, which were indexed to the (111), (200), and (220) planes, respectively. The SnO_2_ with Ag particles had a cassiterite crystal structure (JCPDS card no.: 41-1445). Figure 2c presents SEM images of the synthesized AgSnO_2_. A characteristic SEM image of AgSnO_2_ with bitter melon-like structures was observed. A high-magnification image of the prepared AgSnO_2_ revealed particles with a bitter melon-like shape (Figure 2d). The SEM images support the XRD results because of the strong intra- and intermolecular interactions between nearby AgSnO_2_. Owing to its long bitter melon-like shapes, which also act as a barrier against the transmission of oxygen and water vapor, the AgSnO_2_ sticks to the matrix more effectively. Figure S1 presents energy dispersive X-ray spectroscopy (EDS) maps of AgSnO_2_ prepared by ultrasonic irradiation and coprecipitation. Ag, Sn, and O are indicated by the red, green, and yellow areas, respectively (Figure S1b–d).

### 3.2. Characterization of PBAT copolymer

**Figure 3:**
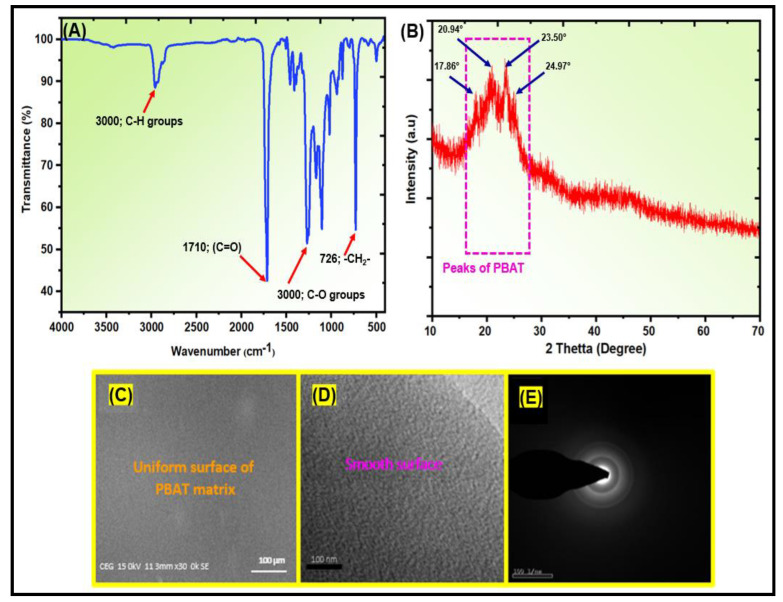
Structural and morphological characterizations of PBAT matrix: (**A**) FTIR; (**B**) XRD; (**C**) SEM; (**D**) TEM; and (**E**) SAED images.

Figure S2 shows the ^1^H-NMR spectrum of the PBAT matrix. The PBAT section had two polymer chains. The signals at 2.34 ppm were attributed to the outer CH_2_ groups in adipic acid, while the signals at 4.11–4.17 ppm were assigned to the outer CH_2_ groups in the 1,4-butanediol unit in the PBAT matrix. The outside CH_2_ groups of the 1,4-butanediol unit were noted at 4.34–4.41 ppm, and the CH in the benzene ring of PBAT was observed at 8.11 ppm. The signal at 3.67–3.75 ppm was attributed to the CH_2_ groups of the 1,4-butanediol next to the –OH group at the end of PBAT. Figure 3A presents the FTIR spectra of the synthesized PBAT matrix. The functional groups of PBAT fulfill the method statements: peak position at 720 cm^−1^ for different or more adjacent methylene (–CH_2_–) groups, peaks at 1710 cm^−1^ for carbonyl groups (C=O) in the ester linkage, at 1266 cm^−1^ for the ester C–O, and at 2965 cm^−1^ for aliphatic and aromatic C–H stretching vibrations. Wavenumbers at 700 and 900 cm^−1^ allow for the observation of the bending peaks of the benzene replacement. Figure 3B presents the XRD patterns of the PBAT polymer. The sample has a broad intensity, with peaks appearing at 17.86°, 20.94°, 23.50°, and 24.97° 2θ, suggesting an amorphous structure. Figure 3C shows SEM images of PBAT. The SEM images were homogeneous and smooth. The PBAT exhibited aggregated molecular structures. The structural morphology of synthetic PBAT was studied by TEM, as shown in Figure 3D. TEM showed that the transparent portion of the images represents the smooth and uniform structure of PBAT. Figure 3E presents the selected area of electron diffraction (SAED) pattern of synthesized PBAT. The SAED pattern of the PBAT matrix indicated an amorphous structure. The heat of fusion for the synthesized PBAT was 17.85 J/g. The Tg was 33.4 °C at a scanning rate of 20 °C/min, and the Tm was 125.38 °C at a scanning rate of 20 °C/min. The results are presented in Figure S3 of the supporting documentation.

### 3.3. Characterization of PBAT/AgSnO_2_ Composites

#### 3.3.1. Morphology and Thickness of the PBAT/AgSnO_2_ Composites

**Figure 4:**
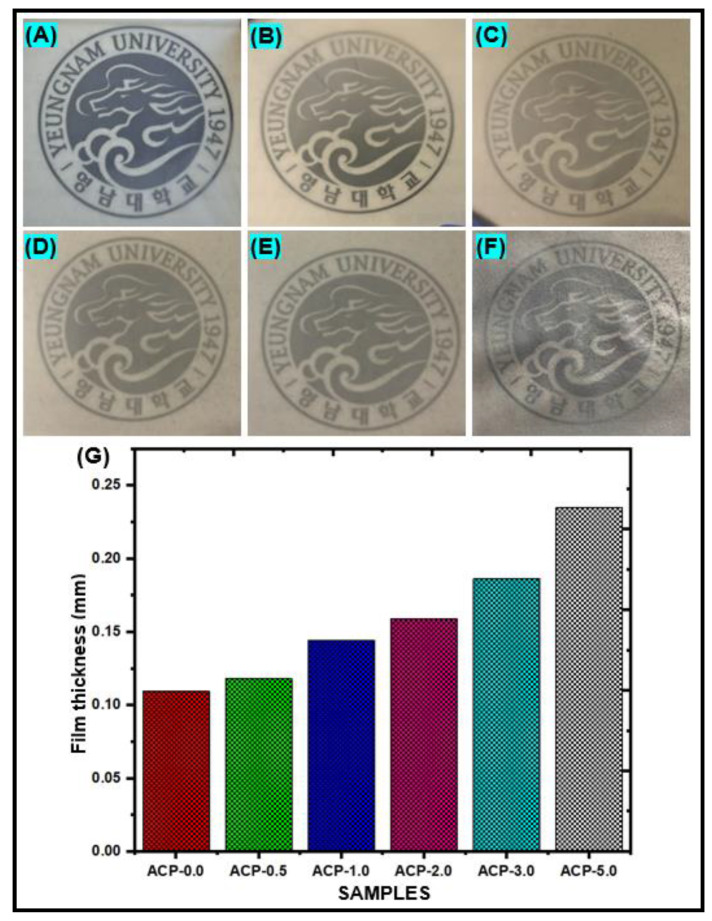
Visual appearance of the PBAT film (**A**) and PBAT composites with 0.5 (**B**), 1.0 (**C**), 2.0 (**D**), 3.0 (**E**) and 5.0 (**F**) wt. % of AgSnO_2_ MPs; (**G**) Film thickness of PBAT/AgSnO_2_ composite films.

The clean PBAT and PBAT/AgSnO_2_ composites with several percentages of (0.5 to 5.0 wt. %) AgSnO_2_ exhibited uniform surfaces because the viscosities of the respective film-forming solutions were suitable for casting a film. The appearance of PBAT/AgSnO_2_ composite films is displayed in [Figure 4A–F]. The PBAT/AgSnO_2_ composite film with 5.0 wt.% AgSnO_2_ (ACP-5.0) used to have a rougher surface because of AgSnO_2_ agglomerations and air bubbles trapped in the casting solution. These flaws occurred because the viscosity of the PBAT solution with 5.0 wt.% AgSnO_2_ prevented air bubbles from exiting and the AgSnO_2_ content above the optimal value (above 3.0 wt.%) enhanced PBAT and metal oxide (AgSnO_2_) interactions. AgSnO_2_ MPs were used to examine the effect of the AgSnO_2_ content on the mechanical and barrier properties of PBAT composites, and the outcomes of these formulations were compared with those of neat PBAT film. Figure 4G shows values for the film thickness of various PBAT and composite films. The film thickness ranged from 0.109 to 0.235 mm. In contrast to the PBAT composite film with 5.0 wt. % AgSnO_2_ (ACP-5.0), which showed a 53.6% thickness, and the neat PBAT film showed an 11.5% thickness. The mechanical and barrier characteristics of the materials are also affected by their strength.

#### 3.3.2. Attenuated Total Reflection Infrared (ATR-IR) Spectroscopy

**Figure 5:**
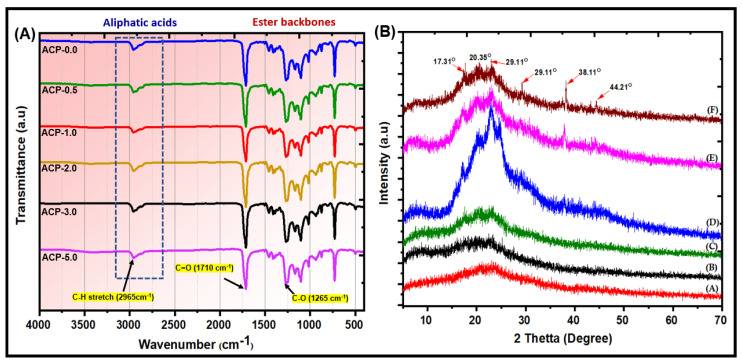
(**A**) ATR-FTIR; (**B**) XRD patterns of the (A) PBAT, and (B) ACP-0.5, (C) ACP-1.0, (D) ACP-2.0, (E) ACP-3.0, and (F) ACP-5.0 composite films.

The ATR-IR spectra of PBAT and PBAT/AgSnO_2_ composites revealed the following peaks. Asymmetric stretching of the aromatic and aliphatic C–H at 2965 cm^−1^, carbonyl extending from the ester bond at 1710 cm^−1^, C–O stretching vibration at 1265 cm^−1^, and carbonyl stretching vibration at 726 cm^−1^ (associated with the rocking vibrations of adjacent methylene [–CH_2_–] groups of the PBAT backbone). This is in line with the previously reported PBAT ATR-IR spectrum [36]. No noticeable differences were observed between the transmission ATR-IR spectra of the composites and those of PBAT and AgSnO_2_ because there was insufficient filler present. The ATR-IR spectra of the composites, which were adjusted to the composition in the first few micrometers, as evidenced by the Ag-O-Sn vibration at 1100 cm^−1^, can detect AgSnO_2_. With the carbonyl carbon of the PBAT acting as an internal reference, the intensity of this point increased with increasing filler concentration, showing that the filler segregates to the film surface. This is more evident with more filler. Figure 5A shows the ATR-IR of the PBAT and the composites (ACP-0.5, ACP-1.0, ACP-2.0, ACP-3.0, and ACP-5.0).

#### 3.3.3. X-ray Diffraction (XRD)

Figure 5B shows XRD patterns for the PBAT and PBAT/AgSnO_2_ composites. PBAT showed the characteristic XRD peaks at 17.31°, 20.35°, and 23.00° 2θ, which were indexed to the (011), (100), and (111) planes, respectively, [37]. All the PBAT/AgSnO_2_ composites showed these peaks at the same 2θ values. The intensities of the PBAT characteristic peaks decreased as the amount of AgSnO_2_ increased, but the reduction was minimal. As a result, after the filler AgSnO_2_ was added, there was no noticeable change in the crystal structure of PBAT. The crystallinity of PBAT in the composites was not significantly affected by AgSnO_2_. AgSnO_2_ indicates the characteristic peaks of approximately 29.11°, 38.11°, and 44.21° 2θ, which PBAT does not depict, under similar situations. The intensity of the peaks, while typical of AgSnO_2_, improved as the wt.% increased, showing that the development of a discrete crystalline phase is often used as a filler in the composite materials. These results indicated that the filler AgSnO_2_ did not affect the PBAT semi-crystal structure.

#### 3.3.4. Scanning Electron Microscopy (SEM)

**Figure 6:**
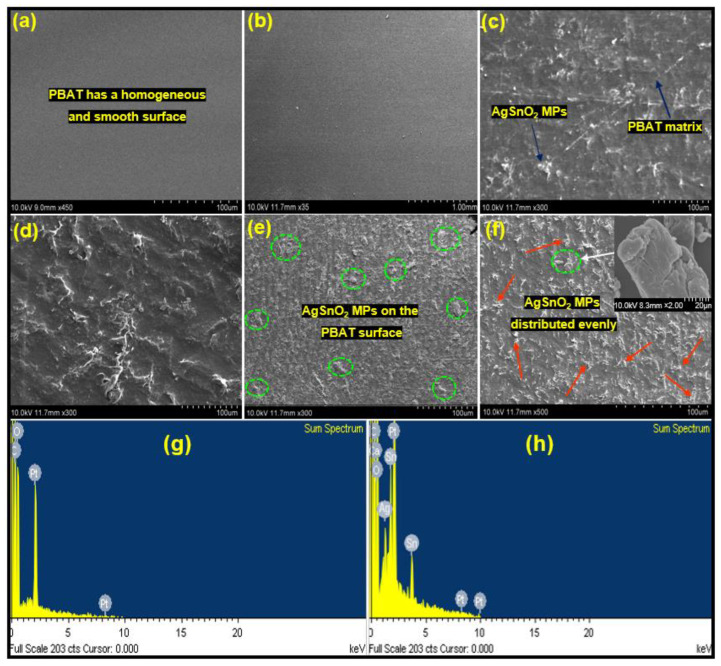
SEM images of the surfaces of PBAT and its composites: (**a**) 0.0, (**b**) 0.5, (**c**) 1.0, (**d**) 2.0, (**e**) 3.0, and (**f**) 5.0 wt. % of AgSnO_2_; (**g**) Energy-dispersive spectrum of PBAT film; (**h**) PBAT with 5.0% AgSnO_2_ MPs.

Figure 6 presents SEM images of the PBAT/AgSnO_2_ composites with various AgSnO_2_ loadings percentages. The figures clearly show that the integration of AgSnO_2_ in the polymer matrix of pure PBAT film had a smooth surface. The surface became increasingly rough as the AgSnO_2_ loading was increased. Figure 6b shows an SEM image of a PBAT/AgSnO_2_ composite film synthesized with the lowest AgSnO_2_ loading of 0.5 wt.% (ACP-0.5). In PBAT, the AgSnO_2_ are distributed evenly in this image. AgSnO_2_ aggregate in the PBAT matrix because of the increased interaction between AgSnO_2_ as its loading was increased. Images for PBAT/AgSnO_2_ composites prepared with a AgSnO_2_ loading of 1.0, 2.0, 3.0, and 5.0 wt.% showed this property. The highest AgSnO_2_ aggregation was observed in the ACP-5.0 composite film. These results suggest that the C=O group of PBAT and AgSnO_2_ have strong bonding interactions in the composite PBAT/AgSnO_2_ composites. SEM was used to identify the nanostructured composite developed between AgSnO_2_ and PBAT. The elemental content of the sample was verified by EDS (Figure 6g,h). AgSnO_2_ in the ACP-5.0 film. According to the spectral data, the composites represent the optimal amount of Ag, Sn, O, and other elements. PBAT and the filler in the ACP-5.0 film should have an atomic composition of Ag and Sn, but EDS revealed 2.30 and 3.75.

#### 3.3.5. X-ray Photoelectron Spectroscopy Analysis (XPS)

**Figure 7:**
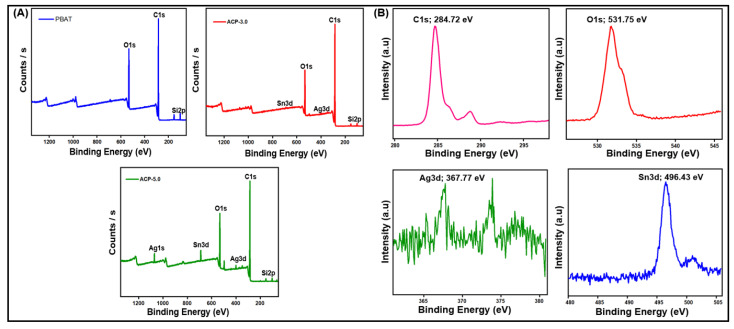
PBAT composite film: (**A**) XPS survey spectrum, and (**B**) high-resolution de-convoluted spectrum of C1s, O1s, Ag3d, and Sn3d.

XPS was used to assess the energy levels of the element levels. According to XPS analysis, the filler was found at the air-film interface. Figure 7A shows the survey spectrum for the PBAT/AgSnO_2_ composite film (the full binding energy range is not shown to view the region of interest). The spectrum confirmed the combination of silver and stannous atoms on the surface. The silver and stannous atom composition would be 6.91 and 5.10, provided the filler is placed into the composite film evenly. ACP-5.0 presents the multiplex spectrum of the C1s, O1s, Ag3d, and Sn3d regions. In Figure 7B, the presence of carbon (C1s) states was observed at 284.72 eV, which corresponds to C–C bonding. In addition, oxygen (O1s) was observed at 531.75 eV, which corresponds to C=O. At 367.77 and 373.85 eV, which corresponds to Ag–O, the incorporation of silver (Ag3d) phase was observed. Furthermore, stannous (Sn3d) state incorporation was observed at 496.43 eV, which is identical to Sn–O. Similar results were observed for PBAT nanocomposites filled with increasing nanoclay contents [38,39]. Ag, Sn, C, and O were found in the survey spectrum.

#### 3.3.6. Thermogravimetric (TGA) Analysis

The thermal stability of PBAT composites with different loadings of 0.5, 1.0, 2.0, 3.0, and 5.0 wt. % AgSnO_2_ was studied by TGA. A heating rate of 20 °C min^−1^ was used to examine the thermal behavior in a N_2_ atmosphere. Figure S4 shows the results of the TGA analysis of the materials, and Table 1 provides an overview of the associated data. The TGA curve for PBAT showed a peak at 416 °C, while the TGA curves for PBAT/AgSnO_2_ composites showed higher peaks was 417.33 °C, 418.20 °C, 419.75 °C, 421.05 °C, and 428.80 °C for 0.5, 1.0, 2.0, 3.0, and 5.0 wt. % AgSnO_2_, respectively (Figure S4A). As a result, TGA revealed improvement in the thermal properties of PBAT/AgSnO_2_ composites over PBAT polymers. The relatively smaller AgSnO_2_ (with a high surface area) ad so more consistent particle dispersion in the PBAT is too responsible for the elevated temperatures for thermal decomposition for PBAT/AgSnO_2_ composites. The AgSnO_2_ effectively inhibits the random chain thermal offers a range of PBAT because of these structures. A higher degradation temperature was also induced by the improvement in the crystallinity of the PBAT matrix after the addition of AgSnO_2_. This result was confirmed by the XRD analysis of the previously presented composites (or enhanced thermal stability). The mechanical characteristics of the materials will need to be examined in much more detail. Although the AgSnO_2_-reinforced composite film has somewhat lower degradation temperatures than the clean PBAT film, it is still used for applications that require food-based sterilization due to its higher thermal stability (up to 279–287 °C) as pure PBAT film.

#### 3.3.7. Differential Scanning Calorimetry (DSC)

Figure S4B presents the crystallization curves of PBAT and PBAT/AgSnO_2_ composite films, and Table 1 summarizes the data. The crystallization characteristics of PBAT and its composites were studied. During 20 °C min^−1^ heating, the Tm of neat PBAT increased slightly from 125.38 to 132.55 °C, while the Tg remained unchanged. The larger thickness and perfection of the polymer are always related to the increase in Tm. On the other hand, a slight change in crystal thickness was noted. Although the basic thermal characteristics of the samples were mostly unchanged, AgSnO_2_ dramatically altered the way PBAT crystallized. When AgSnO_2_ was added to PBAT, the temperature at which crystallization (Tc (onset)) started to develop improved from 91.9 °C for neat PBAT to 110.6 °C for PBAT composites with a 5.0% AgSnO_2_ content. For the PBAT/AgSnO_2_ composite films, the DSC crystallization peak increased, indicating that AgSnO_2_ is a good crystallizing agent for increasing the crystallization temperature peak of PBAT. PBAT with semicrystalline polymers with aromatic rings in their backbones can promote the catalytic action of aromatic chemical structures. For example, the PBAT for aliphatic-aromatic polyester could use carbon nanotubes [40].

#### 3.3.8. Mechanical Strength of PBAT/AgSnO_2_ Composites

**Figure 8:**
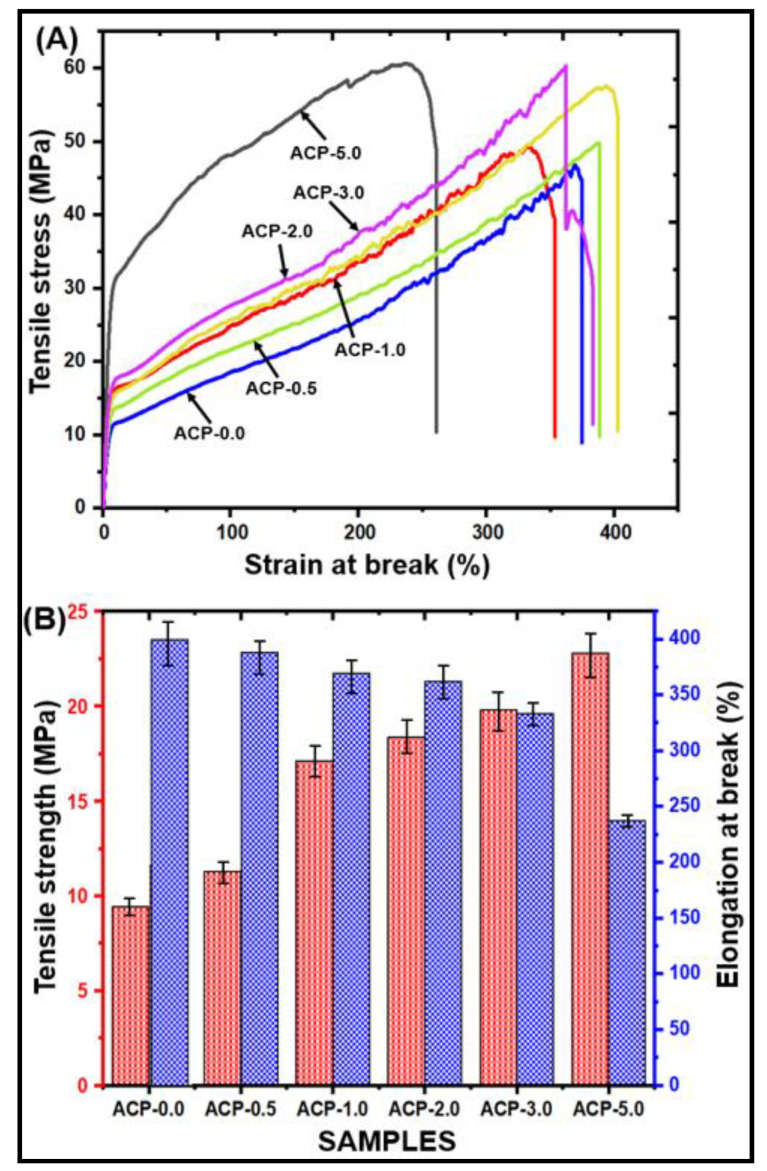
Mechanical strength of PBAT/AgSnO_2_ composites: (**A**) Stress-strain curves; (**B**) Tensile strength vs. Elongation at break values.

The mechanical properties of the PBAT and PBAT/AgSnO_2_ composite films were examined to determine their mechanical performance, as shown in Figure 8. Figure 8A shows the stress-strain curves for PBAT/AgSnO_2_ composites. The tensile strength and elongation at break of the AgSnO_2_-filled PBAT composites are influenced by filler loading, as shown in Figure 8B. Increasing the AgSnO_2_ concentration in the PBAT matrix from 0.5% to 5.0% increased the tensile strength significantly. When compared with pure PBAT (9.43 MPa), the PBAT/AgSnO_2_ (5.0 wt.%) composite film had a higher tensile strength (22.82 MPa). Increased filler concentrations could explain the discontinuity because it weakens the strength, and the modulus is reduced due to the filler-matrix interface and the agglomeration of filler particles. Neat PBAT is highly elastic, with a high elongation at break (394.28%) and a low tensile strength (9.43 MPa). The tensile strength changed significantly with the inclusion of 1.0 wt.% AgSnO_2_ (17.12 MPa), and the elongation at break decreased to 369.15%. An increase in AgSnO_2_ concentration to 5.0 wt.%. resulted in an increase in tensile strength (22.82 MPa) and a decrease in the elongation at break to 237.00%. The AgSnO_2_ filler and PBAT exhibited good interfacial adhesion, which is probably the reason. Strong interfacial bonding is required for success between the matrix and the filler in terms of stress. SEM showed that the filler appears to have a good dispersion, which most likely leads weak areas to form in the composites, which leads the sample to break off. The stiffness and the material ductility are sacrificed to obtain the strength value. When a filler is introduced, the stiffness should increase, but it may burst too soon and lose strength because the filler makes the material less brittle. The improvement in tensile characteristics can be attributed to torrefaction because of compatibility with the PBAT matrix and the proper dispersion of hydrophobic mass. As a result, the tensile properties match the results of the SEM. FTIR spectroscopy suggested that this might be the outcome of filler aggregation. The entanglement density is reduced when the filler content increases. Stress concentration spots are promoted at the macro-phase separated filler-polymer interface. Hence, the tensile strength is increased while still maintaining the characteristically good elongation at break of the unfilled PBAT.

#### 3.3.9. Oxygen Transmission Rate (OTR)

**Figure 9:**
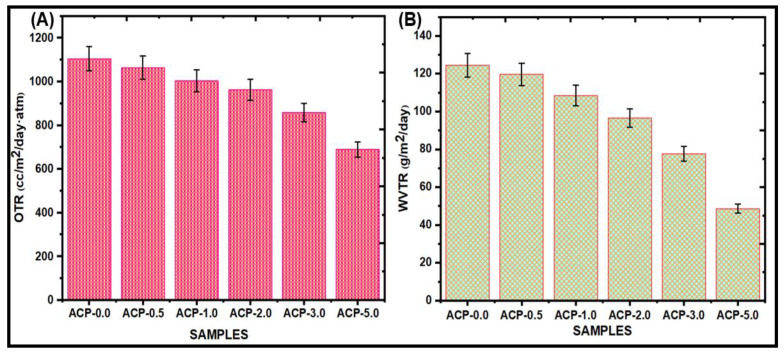
(**A**) OTR; (**B**) WVTR measurements for poly(butylene adipate-*co*-terephthalate) (PBAT) composite films.

Figure 9A presents the OTR of the PBAT and PBAT/AgSnO_2_ composites. For the PBAT film, the OTR was 1104.62 cc/m^2^/day·atm, which decreased to 688.25 cc/m^2^/day.atm after AgSnO_2_ (5.0 wt.%) mixing on PBAT. The addition of a different weight % of AgSnO_2_ then led to a significant decrease. The OTR value was lowered to 1002.58 cc/m^2^/day.atm for 1.0 wt. %; 2.0 wt. % AgSnO_2_ showed the minimum value of 961.15 cc/m^2^/day.atm. The formation of a tortuous path that makes it pass through the film is challenging for gas molecules and is the process behind increased permeability. In addition, the OTR was decreased by the orientation and highest shuck (off) level of AgSnO_2_ in PBAT. The effect of the filler, the crystallization behavior of the polymer composites, and the interaction between the matrix and fillers at contact are some aspects that might increase the barrier properties of the composites. Venkatesan et al. reported lower barrier properties because of poor interfacial adhesion between the PBAT matrix and SnO_2_ [23]. In this study, adding AgSnO_2_ to PBAT decreased the OTR because of the formation of a more tortuous path for diffusing molecules, which allowed it to evade impermeable fillers.

#### 3.3.10. Water Vapor Transmission Rate (WVTR)

Food packaging materials must have a low WVTR to decrease the consumption of water vapor that is transmitted between the food and the atmosphere [41,42]. PBAT and PBAT/AgSnO_2_ composite films were evaluated for their WVTR, as shown in Figure 9B and listed in Table 2. The pure PBAT has a WVTR of 124.54 g/m^2^/day. The WVTR value was decreased to 48.67 g/m^2/^day after AgSnO_2_ (5.0 wt. %) was mixed into the PBAT matrix. By including 1.0 wt. % of AgSnO_2_, the WVTR value of the films was lowered to 108.45 g/m^2^/day. Adding the 2.0 wt. % of AgSnO_2_ to still resulted in a reduction in the WVTR value, which was 96.57 g/m^2^/day. The PBAT film with 3.0 wt. % AgSnO_2_ incorporation showed the minimum value of 77.72 g/m^2^/day. These results showed that an increase in AgSnO_2_ in the PBAT matrix resulted in a reduction in WVTR. These results might be due to the even distribution of AgSnO_2_ throughout the PBAT matrix, which produced a circuitous path for the transmission of water vapor and extended the effective path of diffusion. AgSnO_2_ and PBAT produced hydrogen bonds that enhanced the force of adhesion and limited the passing of water molecules through the film.

#### 3.3.11. Water Contact Angle Analysis (WCA)

**Figure 10:**
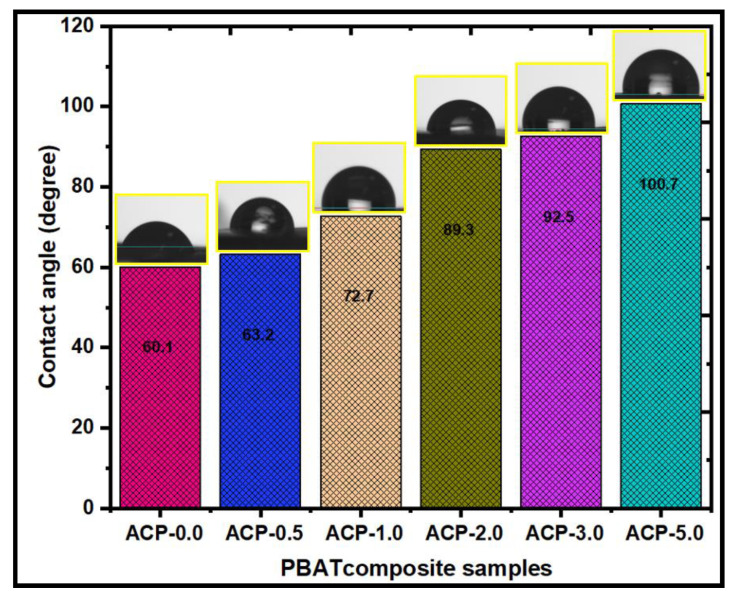
Water contact angle measurements of poly(butylene adipate-co-terephthalate) and its composites.

The wettability was evaluated using the contact angle to find if a surface is hydrophilic or hydrophobic. Hydrophobic surfaces are those with a surface contact angle of more than 100°. Figure 10 shows the contact angle values of the PBAT/AgSnO_2_ composites with the different wt.% of AgSnO_2_. The PBAT film has a 60.1° contact angle. The contact angle value increased to 100.7° when PBAT was mixed with PBAT film, showing the hydrophobicity of the PBAT film. The contact angle value increased to 72.7°, even though PBAT contained 1.0 wt.% AgSnO_2_. Similarly, for 3.0 and 5.0 wt.% of AgSnO_2_ in the PBAT matrix, the contact angle value was increased to 92.1° and 100.7°. The contact angle increased when AgSnO_2_ was added, indicating the hydrophobicity of the PBAT/AgSnO_2_ composite films. The hydrophobicity of AgSnO_2_ was modeled by the density-functional theory. The binding energy between the water molecules was high compared to the absorption coefficient on the AgSnO_2_ surface. As a result, groups of water molecules formed on the surface. In addition, other factors, such as the degree of loading, compatibility, and polymer matrix, have a major impact on hydrophobicity [43].

#### 3.3.12. Soil Degradation of PBAT/AgSnO_2_ Composites

**Figure 11:**
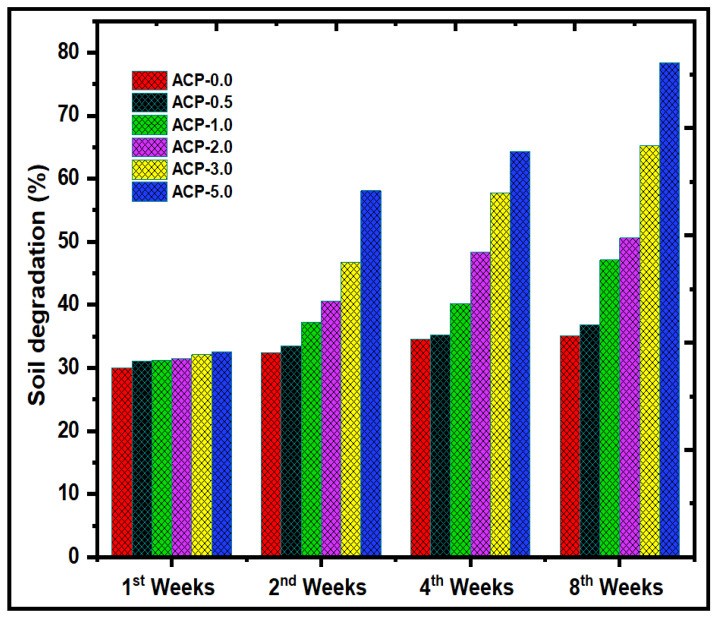
Soil degradation test of PBAT film and PBAT/AgSnO_2_ composite films after 1, 2, 4 and 8 weeks.

The biodegradation of the PBAT/AgSnO_2_ composites was determined by the changes in the weight of the film. The weight loss of the specimens after 1, 2, 4, and 8 weeks was used to evaluate the biodegradation levels of the PBAT/AgSnO_2_ composites, as shown in Figure 11. Figure S5 shows the appearance of the samples. The humidity and chemical structure of the materials have a significant impact on their biodegradability. The PBAT/AgSnO_2_ composite film (ACP-5.0) exhibited the highest % of weight loss after the eighth week owing to its hydrophilicity. The polymer could be quickly absorbed by soil moisture, weakening the polymer chains and making them more susceptible to breakdown at the aliphatic chains. ACP-3.0 degraded to only approximately 65% of its original condition after eight weeks, the lowest amount of all the synthesized films. This suggests that the chemical interactions in the polymer were not readily amenable to biodegradation. Interestingly, compared to the PBAT film, the ACP-3.0 and ACP-5.0 films, which included 3.0 wt.% and 5.0 wt.% of AgSnO_2_, biodegraded faster (approximately 65.2 and 78.4%, respectively, in eight weeks). The composites and mixed materials beat the PBAT matrix in terms of strength because of their mechanical strength and intermolecular interactions. PBAT appears to be less vulnerable to soil microorganisms. These results suggest that AgSnO_2_ was present in ACP-3.0 and ACP-5.0 and had a significant impact on their ability to biodegrade. Furthermore, this behavior was observed in the PBAT composites that contained fillers, such as lignin [44,45], starch [46,47,48], cellulose fiber [49], crystals [50], CaCO_3_ [51,52], and eggshell powder [53].

#### 3.3.13. Evaluation of the Antimicrobial Activity of the PBAT Composites

**Figure 12:**
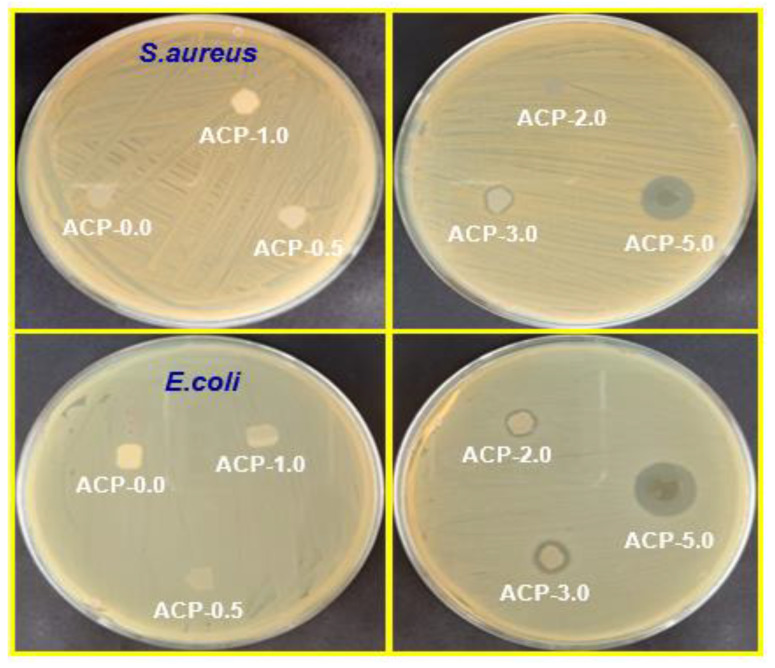
Images of antimicrobial test results of PBAT and its composite films against *S. aureus* and *E. coli* food pathogenic microorganisms.

The major issue of food spoilage and foodborne illnesses is the development of pathogenic microorganisms. Packaging for food must have antimicrobial activity. The antimicrobial effectiveness of the PBAT films was evaluated using the zone of inhibition method on gram-positive and gram-negative bacteria. *S. aureus* and *E. coli* were selected because they are the main microorganisms that lead to foodborne diseases (100 CFU/g total colonies) [54]. The PBAT/AgSnO_2_ composite films showed good antimicrobial activity, as shown in Figure 12, which are represented by the obtained values in Table S2. Food pathogenic microorganisms showed no antimicrobial effects when subjected to the neat PBAT film. The zone of inhibition was increased to 14.20 mm for S. aureus and 16.19 mm for E. coli by adding 5.0 wt.% of AgSnO_2_ to PBAT. Similarly, at 3.0 wt.% of AgSnO_2_, the zone of inhibition was still down to 9.36 mm for *S. aureus* and 12.84 mm for *E. coli*. The 0.5 wt.% of AgSnO_2_ composites showed the highest reduction of 8.11 mm for *S. aureus* and 8.29 mm for *E. coli*. The good antimicrobial activities of AgSnO_2_ were the source of its ability to prevent the growth of gram-negative and gram-positive bacteria. The addition of AgSnO_2_ to the PBAT matrix increased the antimicrobial properties of the composite films.

#### 3.3.14. Migration of the AgSnO_2_ Studies

Table 3 lists the migration of the PBAT and PBAT/AgSnO_2_ composites. Only a limited amount of research has concentrated on the migration of oxide nanoparticles, while the most popular studies on the migration of nanostructured materials from food packaging to foods were focused mainly on nanosilver [55,56]. The requirements for conducting migration tests are specified in the European Commission (EC) regulations. Most migration studies used inert ingredients because of the lack of studies on the migration of particles into actual foods. The level of migration of AgSnO_2_ components (silver and stannic oxide) for the small amounts of material migrated [57] increased mostly with temperature and time during the day. AgSnO_2_ was used in the PBAT composites at different concentrations (0.5 to 5.0 wt.%), and the average concentration was 20.00 mg/L. In conformity with the results of the migration analysis, 10.95 mg/L (SnO_2_ = 407; Ag ˂ 0.27) of AgSnO_2_ migrated from the PBAT composites at a constant temperature of −2 °C. In PBAT at 0 °C, 9.47 mg/L (SnO_2_ = 355; Ag ˂ 0.15) of AgSnO_2_ was migrated. Hence, some AgSnO_2_ leakage occurred in the food samples that were stored. At 0 °C, the PBAT migration was at its maximum level. The maximum migration limit is 0.01 mg/L based on the standard [58].

#### 3.3.15. Food Packaging Experiment

The ability of ACP-5.0 to protect food and extend its shelf life was evaluated using it as a protective film for carrot vegetables. Studies of the physical changes in the carrot vegetable were performed, and the results were compared with those of carrots wrapped in commercial polyethylene for 14 days at room temperature and those left open to the air. In contrast to the current PBAT/AgSnO_2_ composites (ACP-0.5, ACP-1.0, ACP-2.0, and ACP-3.0), and PBAT film, the ACP-5.0 composite film showed a good antimicrobial, mechanical, water vapor, and oxygen barrier properties.

**Figure 13:**
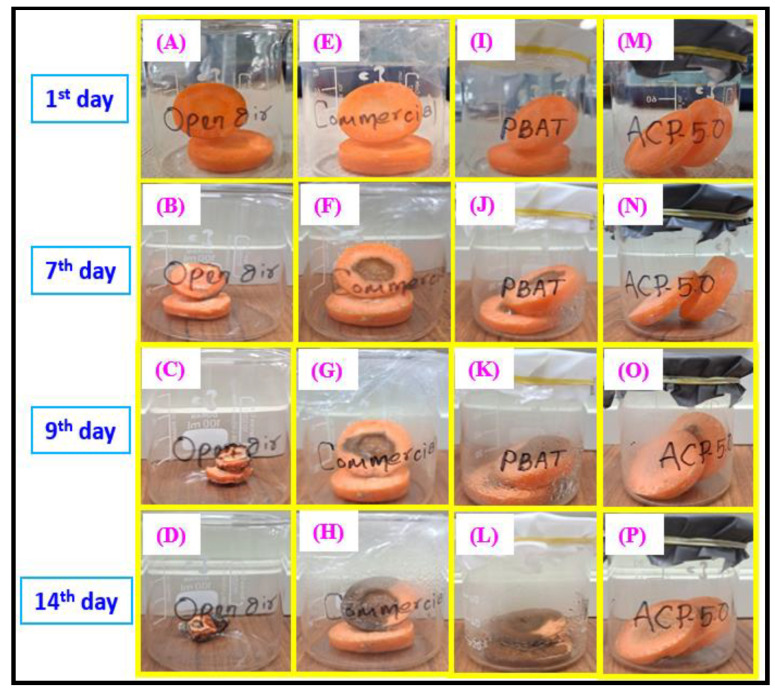
Photograph of food quality test using carrot with commercial LDPE, PBAT and PBAT/AgSnO_2_ composite films during storage at 25 °C.

Figure 13 represents the external appearance of packed carrot slices after 14 days of observation. Compared to the carrots packaged with neat PBAT, carrots packaged with ACP-5.0 film have the finest color and shine [Figure 13I–L]. After three days, the carrots stored in the open [Figure 13A–D] or inside a commercial polyethylene film started to deteriorate and were completely damaged by the 14 days of observation. The ACP-5.0 films [Figure 13M–P] kept the objects fresh, even after nine days, with no discernible deterioration. By the conclusion of the 14th day, the carrots showed a few minor changes, but they were wet and delicious. Indumathi et al., and Chi et al., indicated that the shelf lives of fruit and vegetables were increased by adding nanofillers in biodegradable films used for food preservation [59,60]. When used to package foods, these packaging films can prevent foods from browning while still being stored and increase the duration of food storage. The results suggested that biodegradable PBAT/AgSnO_2_ composites can be used for food packaging.

## 4. Conclusions

The solvent casting method was employed to develop composites containing PBAT as a potential replacement for modern food packaging and wound-healing materials [61]. ATR-IR, XRD, AgSnO_2_, and PBAT appeared to have established extensive interactions according to SEM. The very uniform AgSnO_2_ distribution in the PBAT/AgSnO_2_ composite films was confirmed by the SEM. Compared to the PBAT film, PBAT/AgSnO_2_ composites had good mechanical and soil degradation characteristics. With 5.0 wt.% (22.82 MPa) of filler loaded, the tensile strength increased to its maximum, while 0.5 wt.% (11.25 MPa) of filler was sufficient to double the strength. With increasing AgSnO_2_ concentration in PBAT, the WVTR of the composite films decreased from 124.54 g/m^2^/day to 48.67 g/m^2^/day. The lower OTR was observed for ACP-5.0, which was 688.25 cc/m^2^/day.atm. The OTR values decreased by approximately 37.69% for PBATs loaded with 5.0 wt.% AgSnO_2_. The water contact angle of PBAT increased from 72.7° to 100.7° as the AgSnO_2_ content was increased, which was accompanied by an increase in hydrophobicity. The antimicrobial results showed that PBAT/AgSnO_2_ (5.0 wt.% of AgSnO_2_) inhibited the growth of *E. coli* and *S. aureus*. This study shows that PBAT/AgSnO_2_ composite film (ACP-5.0) degraded in a specific soil over eight weeks. Furthermore, the microorganisms in the soil actively involved in the biodegradation of PBAT were identified and analyzed. In addition, the packaging study for carrot vegetables revealed that the composites had good rot-inhibiting and fresh food product characteristics. The results showed that the PBAT/AgSnO_2_ composites had many improved physical and mechanical characteristics and a low OTR and WVTR. The use of PBAT films as a food packaging material for a variety of safe foods is strongly recommended.

## Figures and Tables

**Table 1:** TGA and DSC values of the PBAT and PBAT/AgSnO_2_ composite films.

S. No	Samples	TGA	DSC		Ash Content (%) ^b^
		Final Degradation Temperature (°C) ^a^	Tg (°C)	Tm (°C)	
1.	ACP-0.0	416.00	−33.2	125.38	6.20
2.	ACP-0.5	417.33	−34.0	127.50	5.91
3.	ACP-1.0	418.20	−35.4	128.45	5.85
4.	ACP-2.0	419.75	−36.9	129.81	5.50
5.	ACP-3.0	421.05	−38.2	130.24	5.24
6.	ACP-5.0	425.80	−40.7	132.55	4.75

^a^ Temperature at which the final mass loss was recorded. ^b^ Mass percentage of material remaining after TGA at a maximum temperature of 700 °C.

**Table 2:** Barrier properties of PBAT film and PBAT/AgSnO_2_ composite films.

S. No	Composition of PBAT/AgSnO_2_ Composites (wt.%)	OTR (cc/m^2^/day.atm)	WVTR (g/m^2^/day)
1.	100/0.0	1104.62 ± 3.40 ^a^	124.54 ± 2.96 ^a^
2.	99.5/0.5	1062.59 ± 2.01 ^a^	119.59 ± 2.28 ^a^
3.	99.0/1.0	1002.58 ± 2.74 ^c^	108.45 ± 3.15 ^c^
4.	98.0/2.0	961.15 ± 3.05 ^a^	96.57 ± 2.71 ^c^
5.	97.0/3.0	857.62 ± 2.55 ^b^	77.72 ± 3.40 ^a^
6.	95.0/5.0	688.25 ± 1.81 ^c^	48.67 ± 2.20 ^a^

^a–c^: Different letters within the same column indicate significant differences among film samples (*p* < 0.05).

**Table 3:** Studies on the migration of AgSnO_2_ mixed to PBAT packaging materials.

Concentration in Packaging Materials	Matrix	Test Procedure	Analytical Methods	Migration Amount (mg/L)
AgSnO_2_ (20.0 mg/L)	PBAT film	The composite was exposed to the simulant for 2 h at −2 °C.	ICP-MS	SnO_2_ = 407 Ag < 0.21
	PBAT film	The composite was exposed to the simulant for 2 h at 0 °C.	ICP-MS	SnO_2_ = 355 Ag < 0.15

## Data Availability

Not applicable.

## Supplementary Materials

The following supporting information can be downloaded at: https://www.mdpi.com/article/10.3390/polym15030554/s1, Figure S1: EDS images of AgSnO_2_ microparticles; Figure S2: ^1^H-NMR spectrum of PBAT; Figure S3: DSC curve of PBAT; Figure S4: TGA (A); DSC curves (B), of PBAT/AgSnO_2_ composite films: (A) ACP-0.0, (B) ACP-0.5, (C) ACP-1.0, (D) ACP-2.0, (E) ACP-3.0, and (F) ACP-5.0; Figure S5: Images of PBAT film and PBAT/AgSnO_2_ composites after burial in soil for 1, 2, 4 and 8 weeks; Table S1: Compositions of PBAT/AgSnO_2_ composites prepared and their nomenclature; Table S2: Antimicrobial activity of PBAT/AgSnO_2_ composites against *S. aureus* and *E. coli*.
