# Supplementary material for: Preparation and Performance of Biodegradable Poly(butylene adipate-co-terephthalate) Composites Reinforced with Novel AgSnO2 Microparticles for Application in Food Packaging
Source: Polymers (Basel). 2023 Jan 21;15(3):554. doi: 10.3390/polym15030554 (PMC9921653; doi:10.3390/polym15030554)
Supplement: Supplementary file 1 [file polymers-15-00554-s001.zip › polymers-2155374-supplementary.pdf]

Supplementary data

Figure S1. EDS images of $AgSnO_2$ microparticles

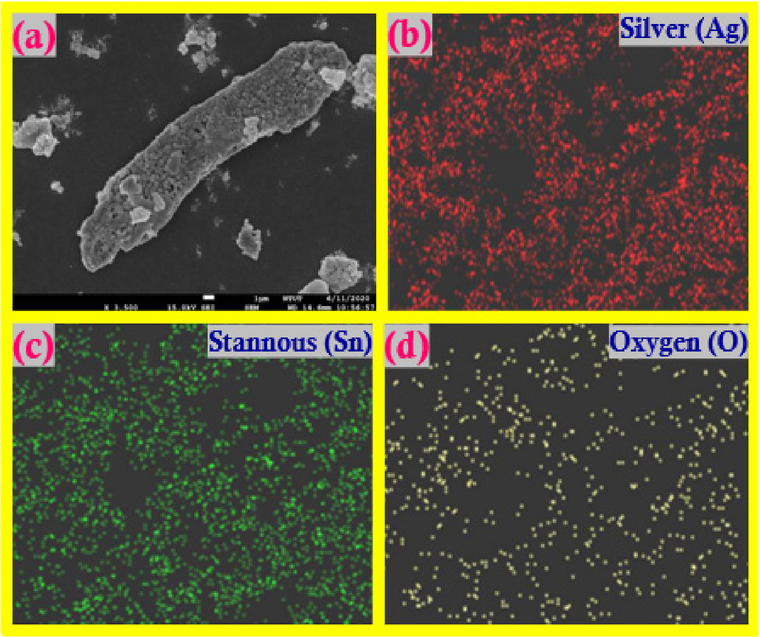

Figure S2. ^{1}H-NMR spectrum of PBAT

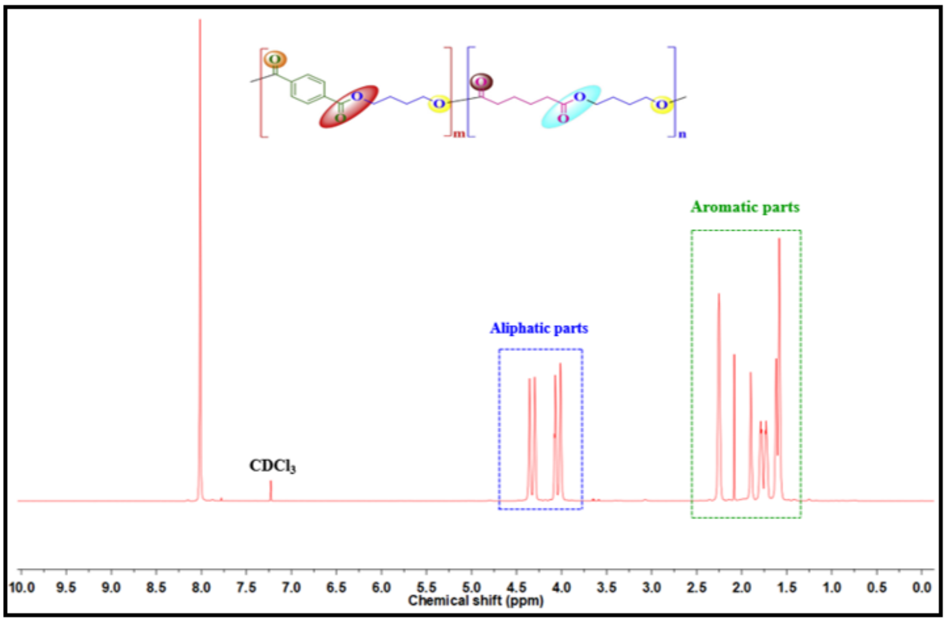

Figure S3. DSC curve of PBAT

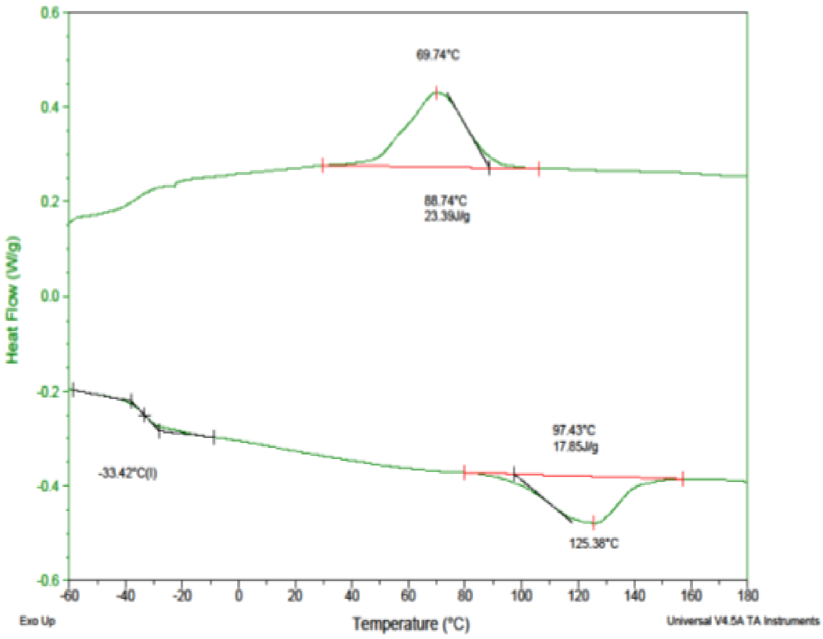

Figure S4. TGA curves (A); DSC curves (B), of PBAT and PBAT/$AgSnO_2$ composite films: (A) ACP-0.0, (B) ACP-0.5, (C) ACP-1.0, (D) ACP-2.0, (E) ACP-3.0, and (F) ACP-5.0.

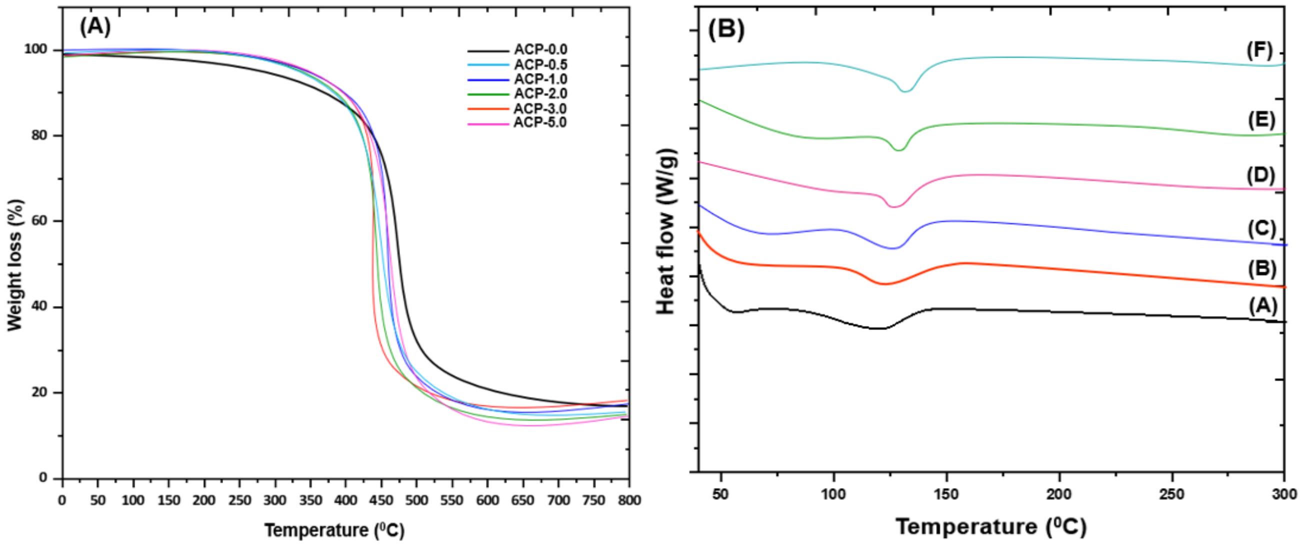

Figure S5. Images of PBAT film and PBAT/AgSnO2 composites after burial in soil for 1, 2, 4 and 8 weeks

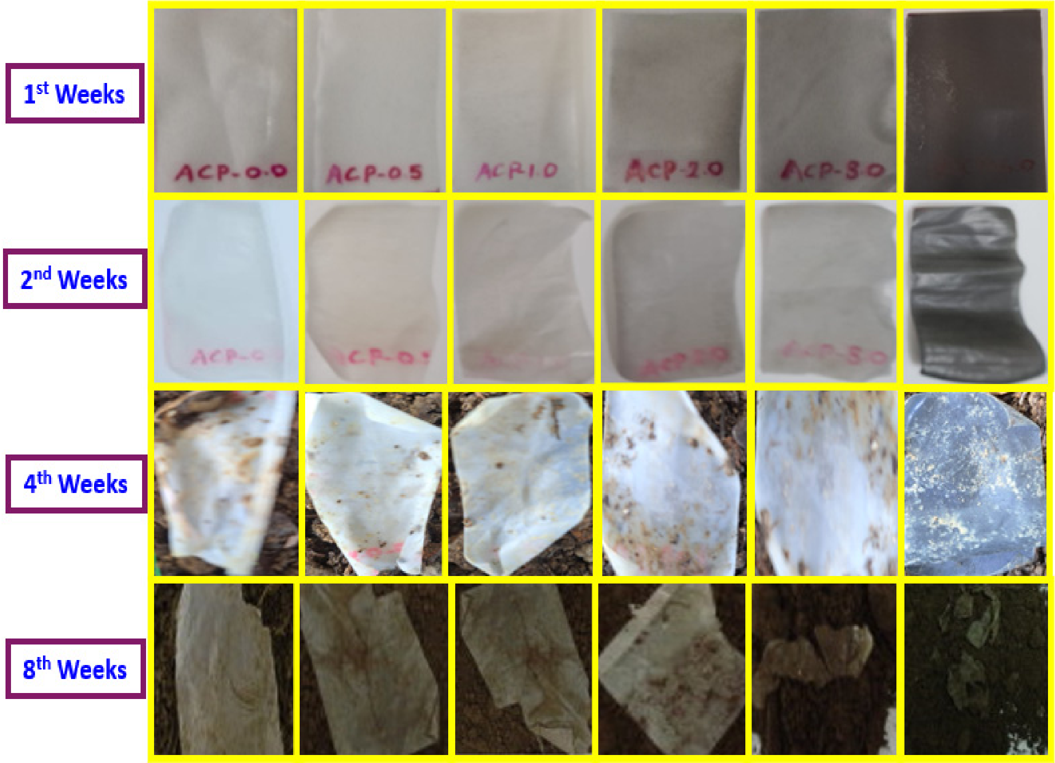

Table S1. Compositions of PBAT/$AgSnO_2$ prepared and their nomenclature

S. No.	Nomenclature	PBAT (g)	$AgSnO_2$ microparticles (mg)	
			Percentages of PBAT	MPs (mg)
1.	ACP-0.0	2.0	2.00	00.0
2.	ACP-0.5	2.0	1.99	10.0
3.	ACP-1.0	2.0	1.98	20.0
4.	ACP-2.0	2.0	1.96	40.0
5.	ACP-3.0	2.0	1.94	60.0
6.	ACP-5.0	2.0	1.90	100.0

Table S2. Antimicrobial activity of PBAT/$AgSnO_2$ composites against *S. aureus* and *E. coli*

Strain	Zone of inhibition in (mm)					
	ACP-0.0	ACP-0.5	ACP-1.0	ACP-2.0	ACP-3.0	ACP-5.0
S. aureus	8.00 ± 3.84^{c}	8.11 ± 3.84^{b}	8.45 ± 2.35^{b}	8.77 ± 1.85^{c}	9.36 ± 2.63^{a}	14.20 ± 2.56^{a}
E. coli	8.00 ± 1.51^{b}	8.29 ± 2.25^{a}	8.62 ± 2.78^{c}	9.13 ± 3.01c	12.84 ± 3.40^{b}	16.19 ± 4.05^{c}

Results are quoted as the mean ± standard deviation of three replicates. a-c: Different letters within the same column indicate significant differences among film samples ($p < 0.05$).
